# *In vitro* evaluation of the composition and acaricidal efficacy of *Urtica fissa* leaf ethyl acetate extract against *Sarcoptes scabiei* mites

**DOI:** 10.17221/6/2023-VETMED

**Published:** 2023-05-24

**Authors:** Fei Liao, Taotao Bao, Guangyao Tao, Yanchun Hu, Changquan Han

**Affiliations:** ^1^Key Laboratory of Animal Disease and Human Health of Sichuan Province, College of Veterinary Medicine, Sichuan Agricultural University, Wenjiang, P.R. China; ^2^Guizhou Vocational College of Agriculture, Qingzhen, P.R. China; ^3^Qiandongnan Center for Animal Disease Control and Prevention, Kaili, Guizhou, P.R. China

**Keywords:** acaricidal properties, LC-MS, sarcoptic mange, *Urtica fissa*

## Abstract

In veterinary medicine, natural products provide an alternative to chemical agents for mite management. In the present study, the acaricidal efficacy of *Urtica fissa* leaf ethyl acetate extract against *Sarcoptes scabiei* mites was examined. The chemical composition of the extract was determined using liquid chromatography-mass spectrometry (LC-MS) analysis. The ethyl acetate extract was found to be extremely toxic to mites at a concentration of 100 mg/ml (m/v), killing all *S. scabiei* within two hours. The median lethal time (LT50) values for ethyl acetate extract concentrations of 25, 50, and 100 mg/ml against *S. scabiei* were 1.706, 1.204, and 0.750 h, respectively. The median lethal dosage (LC50) for *S. scabiei* was 19.14 mg/ml at two hours. The chemical composition of the ethyl acetate extract was evaluated using LC-MS, showing that the major components were schaftoside (8.259%), carnosol (6.736%), prostaglandin A2 (5.94%), 13(*S*)-HpOTrE (4.624%), nandrolone (4.264%), 1*H*-indole-3-carboxaldehyde (4.138%), 9-oxoODE (3.206%), and stearidonic acid (2.891%). In conclusion, these findings indicate that *Urtica fissa* contains promising new acaricidal compounds capable of successfully controlling animal mites.

Sarcoptic mange, a skin condition caused by the mite *Sarcoptes scabiei*, can decimate wild animal populations (Montecino-Latorre et al. 2019). These ectoparasitic infestations have been observed to be highly contagious and often fatal in rabbits (Singh et al. 2022). The mite can also survive off-host for some time while remaining contagious. As the mite has no free-living growth stages or intermediate hosts in its life cycle, its morphology and ecology are well adapted to living in close proximity to the host. As a result, this infectious, host-specific, burrowing, and astigmatic mite acts as an obligatory parasite throughout its life cycle (Pasipanodya et al. 2021). As shown by Lynar et al. (2017), the mites secrete complement inhibitors into burrows, potentially promoting the development of secondary staphylococcal and streptococcal infections and, subsequently, invasive bacterial infections. Ivermectin, avermectin, and other pharmacological drugs are used frequently in veterinary clinics to treat and control psoriasis and have produced positive therapeutic benefits (Liao et al. 2014). However, due to improper drug usage, together with drug resistance and treatment failure, the management of mange mites remains a very difficult task (Gopinath et al. 2018). Therefore, there is an urgent need to develop a new generation of effective acaricides that can simultaneously kill all developmental stages of the scabies mite (adult, nymphal, larval, and egg), which would improve the effectiveness and efficiency of treatment and reduce the rate of scabies recurrence.

Plant-derived acaricides are preferred due to their various modes of action, low cost, simplicity of manufacture in countries with few manufacturing units, minimal non-target impact, high efficiency, and environmental friendliness (Benelli et al. 2017). The stinging nettle, *Urtica fissa,* grows widely in China. There are about 35 *Urtica* species in the world, of which 23 are found in China, mainly distributed in the northern and southwestern regions of the country (Wang et al. 2022). Most *Urtica* species have medicinal properties and have long been used in traditional medicine. In traditional Chinese medicine, *Urtica* extracts have the effects of expelling wind, dredging collaterals, activating blood circulation, and relieving pain and are mainly used to treat rheumatic pain, infant convulsions, and postpartum convulsions, among other applications. Western herbalists have also used stinging nettle extracts, juice, tea, and freeze-dried products, for instance, as blood-replenishing tonics and for the treatment of periodic rhinitis (Upton 2013). Studies have shown that *Urtica* leaves contain flavonoids, lignans, coumarins, triterpenoids, steroids, alkaloids, polysaccharides, organic acids, and other chemical components (Li et al. 2009; Wang et al. 2016; Wang et al. 2018). Studies have found that *U. fissa* plants have a wide range of pharmacological activities, and have been used to treat hyperglycaemia (Mao et al. 2020), as well as having antioxidant (Wang et al. 2019), lipid-regulatory, antinociceptive, anti-inflammatory (Hajhashemi and Klooshani 2013), antibacterial, antitumor, and anti-prostatic hyperplasia (Zhang et al. 2022) effects.

However, to the best of our knowledge, the acaricidal activity of *U. fissa* has not yet been investigated. Here, we assessed the effectiveness of ethyl acetate extracts of *U. fissa* against *S. scabiei in vitro* and investigated the main active components of the extract.

## MATERIAL AND METHODS

### Ethical statement

This study was reviewed and approved by the Animal Ethics and Welfare Committee of Sichuan Agricultural University (Approval No.: 2012-024). Institutional ethical and animal care guidelines were adhered to during sampling, and all procedures were conducted in accordance with the China Guide for the Care and Use of Laboratory Animals. Furthermore, all procedures involving animals and their care were conducted in accordance with ARRIVE guidelines [PLoS Bio 8(6), e1000412,2010] and all plant experiments and collections were done with relevant permissions and complied with the relevant institutional, national, and international guidelines and legislation.

### Plant material and preparation of extracts

Fresh plant material was harvested from Zunyi (27°38'N; 107°46'E, 754 m), Guizhou Province, Southwest China, in July 2022. The *U. fissa* specimens were identified at the College of Veterinary Medicine, Sichuan Agricultural University, P.R. China. The *U. fissa* leaves were dried and crushed in a knife mill. Subsequently, 0.2 kg of the plant material was immersed in 75% ethanol (2 l of 75% per 100 g of plant material) for ultrasonic extraction at 50 °C and 500 W for 1 hour. The extracts were then mixed and concentrated by evaporation using a rotary evaporator. Extractions were performed three times using the same volume of ethyl acetate. After concentration on the rotary evaporator, the extracts were vacuum-dried for 24 h at 60 °C to guarantee thorough elimination of any remaining ethyl acetate that could impact the experimental outcomes.

### LC-MS analysis of extracts

#### LIQUID CHROMATOGRAPHY CONDITIONS

LC was performed on a Vanquish UHPLC System (Thermo Fisher Scientific, Waltham, MA, USA) using an ACQUITY UPLC^®^ HSS T3 (150 × 2.1 mm, 1.8 μm) column; Waters, Milford, MA, USA). The temperature in the column was kept constant at 40 °C. The injection volume and flow rate were set at 0.25 ml/min and 2 μl, respectively. The mobile phases for LC-ESI (+)-MS analysis were (A2) 0.1% formic acid in water (v/v) and (B2) 0.1% formic acid in acetonitrile (v/v). The following gradient was used for separation: 0–1 min, 2% B2; 1–9 min, 2–50% B2; 9–12 min, 50–98% B2; 12–13.5 min, 98% B2; 13.5–14 min, 98–2% B2; 14–20 min, 2% B2. The analytes for LC-ESI (–)-MS analysis were (A3) ammonium formate (5 mM) and (B3) acetonitrile. The following gradient was used for separation: 0–1 min, 2% B3; 1–9 min, 2–50% B3; 9–12 min, 50–98% B3; 12–13.5 min, 98% B3; 13.5–14 min, 98–2%, and B3; 14–17 min, 2% B3 (Zelena et al. 2009).

#### MASS SPECTROMETRY CONDITIONS

Metabolites were detected by mass spectrometry on a Q Exactive (Thermo Fisher Scientific, Waltham, MA, USA) with an ESI ion source. We employed simultaneous MS1 and MS/MS capture (full MS-ddMS2 mode, data-dependent MS/MS). The following parameters were used: spray voltage, 3.50 kV and –2.50 kV for ESI(+) and ESI(–), respectively; sheath gas pressure, 30 arb; aux gas flow, 10 arb; MS1 range, m/z 100–1 000; MS1 resolving power, 70 000 FWHM; the number of data-dependent scans per cycle, 10; MS/MS resolving power, 17 500 FWHM; normalized collision energy, 30 eV; automatic dynamic exclusion time (Want et al. 2013).

### Isolation of *S. scabiei* from rabbits

Institutional ethical and animal care standards were followed during the sampling exercise, and all procedures were performed following the Guidelines for the Care and Use of Laboratory Animals. Scabs obtained from the infected legs of naturally infected rabbits were used to isolate *S. scabiei*. Scabs were placed on Petri dishes and cultured in an incubator for 30 min at 35 °C*.*

To ensure consistency of the results, only recently fed adults were used; this was assessed by the colour of the mites; the darker ones were assumed to have fed more recently than paler individuals (Alimi et al. 2021). The rabbits were given treatment immediately after the materials had been collected.

### Acaricidal activity of extracts *in vitro*

A contact toxicity bioassay was conducted as previously described (Nong et al. 2013) with some modifications. The ethyl acetate extracts were diluted in 10% glycerine to concentrations between 25 and 100 mg/ml (25, 50, 100 mg/ml). Filter paper chips were used to absorb the liquid after pouring the 0.1-ml sample into the Petri dishes (5 cm in diameter and 2 cm high), and 10 mites were introduced to each dish. Liquid paraffin was used as a negative control and ivermectin (1%) as a positive control. Six replicates of each concentration of extract were used. All plates were incubated for 0.5, 1, 1.5, and 2 h at 25 °C with 75% relative humidity before using evaluation under a stereomicroscope. A needle was used to periodically stimulate mites to test their vitality; if no response was observed, the mites were recorded as dead.

### Statistical analyses

SPSS v20.0 (IBM Corp., Armonk, NY, USA) was used for all calculations (Zhou et al. 2019b). The significance of changes in mean mite mortality between various concentrations was determined using the probability technique. The complementary log-log (CLL) model was used to determine the median lethal concentration (LC50) values and median lethal time (LT50) (Liao et al. 2014).

## RESULTS

### Chemical composition of extracts

**Figure 1:**
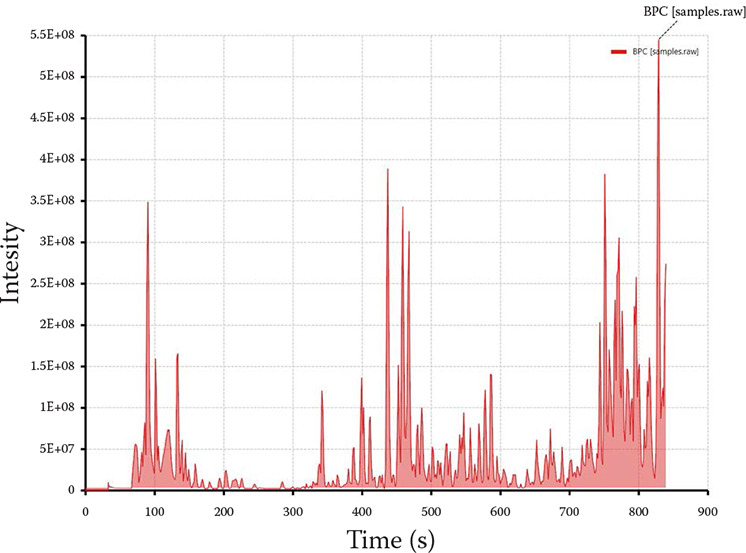
Ion flow diagram extracted from LC-MS/MS samples

A total of 275 mg of ethyl acetate extract was obtained from 200 g of nettle leaves. The extract had an extraction rate of 0.138%. After pre-treat-ment, 886 peaks representing low molecular weight metabolites were identified in the extracts LC-MS/MS (Figure 1). These mostly included low molecular weight organic acids, amino acids, sugars, lipids, sugar alcohols, phenolic compounds, alkaloids, and flavonoids. Schaftoside (8.259%), carnosol (6.736%), prostaglandin A2 (5.94%), 13(*S*)-HpOTrE (4.624%), nandrolone (4.264%), 1*H*-indole-3-carboxaldehyde (4.138%), 9-oxoODE (3.206%), and stearidonic acid (2.891%) were the most abundant components found in the extracts (Table 1).

**Table 1:** Chemical composition of ethyl acetate extract from *U. fissa* leaves

ID	Compound	Mz	Rt (s)	Formula	Relative content (%)
M565T468_2	Schaftoside	565.150 9	467.8	C_26_H_28_O_14_	8.259
M331T796	Carnosol	331.187 1	796.3	C_20_H_26_O_4_	6.736
M335T812_2	Prostaglandin A2	335.219 6	812.4	C_20_H_30_O_4_	5.947
M293T821	13(*S*)-HpOTrE	293.211 3	820.7	C_18_H_30_O_4_	4.624
M275T800	Nandrolone	275.200 0	800.3	C_18_H_26_O_2_	4.264
M146T587	1*H*-indole-3-carboxaldehyde	146.061 1	587.3	C_9_H_7_NO	4.138
M295T812	9-oxoODE	295.225 6	812.4	C_18_H_30_O_3_	3.206
M277T812	Stearidonic acid	277.215 7	812.4	C_18_H_28_O_2_	2.891
M104T86	Gamma-aminobutyric acid	104.107 1	85.6	C_4_H_9_NO_2_	2.282
M565T468_1	Pelargonidin 3-*O*-beta-d-sambubioside	565.138 2	467.8	C_26_H_29_O_14_	1.806
M353T701	(13E)-11a-Hydroxy-9,15-dioxoprost-13-enoic acid	353.229 7	701.2	C_20_H_32_O_5_	1.712
M329T777	Methotrimeprazine	329.172 1	777.2	C_19_H_24_N_2_OS	1.667
M307T438_2	Bz-Arg-OEt	307.175 7	438.5	C_15_H_22_N_4_O_3_	1.386
M273T652_2	Neocembrene	273.256 6	652.3	C_20_H_32_	1.366
M205T399	l-Tryptophan	205.096 3	399.1	C_11_H_12_N_2_O_2_	1.241
M433T541	Genistin	433.107 6	541	C_21_H_20_O_10_	1.237
M313T751	9,12,13-TriHOME	313.237 1	750.9	C_18_H_34_O_5_	1.119
M595T503_2	Cyanidin 3-*O*-rutinoside	595.161 1	503	C_27_H_31_O_15_	1.113
M349T765	Enalaprilat	349.175 6	765	C_18_H_24_N_2_O_5_	1.062
M118T581	Indole	118.065 7	581.3	C_8_H_7_N	0.988
M132T77	l-aspartic acid	132.028 7	77.4	C_4_H_7_NO_4_	0.923
M149T497	Anethole	149.095 5	496.8	C_10_H_12_O	0.872
M303T470	*N*-acetylaspartylglutamate	303.089 2	469.8	C_11_H_16_N_2_O_8_	0.779
M579T520_2	Vitexin 2''-*O*-beta-l-rhamnoside	579.170 2	519.6	C_27_H_30_O_14_	0.697
M235T741	Confertifolin	235.172 1	740.8	C_15_H_22_O_2_	0.689
M137T474	3-hydroxybenzoic acid	137.021 4	474.4	C_7_H_6_O_3_	0.652
M329T781	Labetalol	329.187 0	781.3	C_19_H_24_N_2_O_3_	0.635
M162T567	Indole-3-carboxylic acid	162.054 0	567.3	C_9_H_7_NO_2_	0.590
M175T114	Guanidinosuccinic acid	174.954 6	113.7	C_5_H_9_N_3_O_4_	0.589
M296T812	Alpha-dimorphecolic acid	296.230 5	812.4	C_18_H_32_O_3_	0.585
Total		–	–	–	64.052

Mz = mass to charge ratio; Rt = retention time

### Acaricidal activity of the extracts

**Figure 2:**
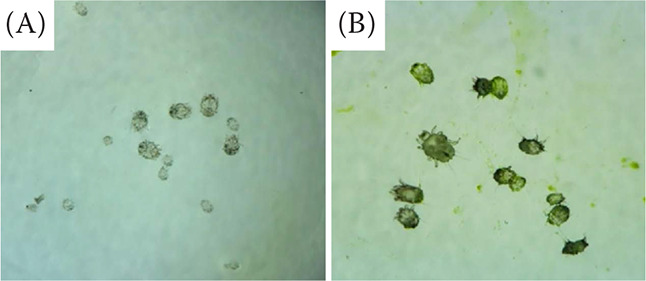
Dead *S. scabiei* (A) Observed by microscopy (× 400) following treatment with ivermectin after 2 hours; (B) Observed by microscopy (× 400) following treatment with ethyl acetate extract of *U. fissa* leaves after 2 hours

The acaricidal efficacy of the *U. fissa* ethyl acetate extracts against *S. scabiei* was evaluated *in vitro*. The results showed that acaricidal activity depended significantly on the extract concentration. The mortality rates of mites treated with the highest concentration of extract (100 mg/ml) and those in the positive control group reached 100% within 2 hours (Figure 2). Significant effects (*P* < 0.05) on *S. scabiei* were also observed over a range of dosages, with 100.0%, 76,7%, and 60.0% of mites killed after treatment with 100, 50, and 25 mg/ml of extract, respectively, within 2 hours (Table 2). Tables 3 and 4 illustrate the lethal concentrations and times for the killing of *S. scabiei*. The toxicity of the ethyl acetate extracts to mites was both concentration- and time-dependent. The LC50 result for *S. scabiei* was 19.14 mg/ml after 2 hours.

**Table 2:** The acaricidal activity of the extract against *S. scabiei in vitro*

Concentration	Time (hours)			
	0.5 mean mortality (%) ± SD	1 mean mortality (%) ± SD	1.5 mean mortality (%) ± SD	2 mean mortality (%) ± SD
25 mg/ml	16.7 ± 5.77^aA^	26.7 ± 15.28^abA^	43.3 ± 11.55^bcA^	60 ± 10.00^cA^
50 mg/ml	23.3 ± 15.28^aA^	43.3 ± 5.77^abA^	60.0 ± 10.00^bcB^	76.7 ± 11.55^cB^
100 mg/ml	43.3 ± 5.77^aB^	66.7 ± 20.82^bB^	80.0 ± 10.00^bcC^	100.0 ± 0.00^cC^
Positive control	83.3 ± 5.77^aC^	100.0 ± 0.00^bC^	100.0 ± 0.00^bD^	100.0 ± 0.00^bC^
Untreated control	0.0 ± 0.00^aD^	0.0 ± 0.00^aD^	3.3 ± 5.77^aE^	6.7 ± 5.77^aD^

In each line, different lowercase letters indicate significant differences in the same treatment concentrations at different time intervals (*P* < 0.05). In each column, different capital letters indicate significant differences in the same time intervals at different treatment concentrations (*P* < 0.05)

SD = standard deviation

**Table 3:** The probit regression analysis of toxicity (LT50) of the extract against mites *in vitro*

Concentration	Regression line	LT50 (h) 95% FL)	Pearson Chi-square
25 mg/ml	y = –1.412 + 0.828x	1.70 (1.413 ~ 2.303)	4.772
50 mg/ml	y = –1.171 + 0.956x	1.226 (0.942 ~ 1.498)	5.213
100 mg/ml	y = –1.116 + 1.489x	0.750 (0.471 ~ 0.934)	8.954

FL = fiducial limits

**Table 4:** Probit regression analysis of toxicity (LC50) of the extract against mites *in vitro*

Time (h)	Regression line	LC50 (mg/ml) (95% FL)	Pearson Chi-square
0.5	y = –2.074 + 0.018x	115.110 (84.362 ~ 379.800)	3.345
1.0	y = –1.479 + 0.022x	66.892 (45.117 ~ 100.326)	6.659
1.5	y = –0.465 + 0.013x	34.973 (19.643 ~ 55.368)	3.265
2.0	y = –0.553 + 0.029x	19.14 (16.887 ~ 31.573)	3.417

FL = fiducial limits

## DISCUSSION

To the best of our knowledge, the above results present the first demonstration of the acaricidal effects of ethyl acetate extracts of *U. fissa* against *S. scabiei*. The toxicity of the extract was both time- and concentration-dependent (Table 2). Comparable *in vitro* and *in vivo* effects have been observed for *Eupatorium adenophorum* ethanol extracts against *S. scabiei* and *Psoroptes cuniculi* (Hu et al. 2014; Liao et al. 2014) whereas an investigation of the effects of *Ligularia virgaurea* extracts against *S. scabiei* found that concentrations of 2 g/ml killed all *S. scabiei* within 2 h and concentrations of 1 g/ml killed all *S. scabiei* within 6 hours (Luo et al. 2015). *Acacia nilotica* ethanol extracts were found to have an LC50 value of 0.218 g/ml at 6 h against *S. scabiei* (Khan et al. 2022), and *Laurus nobilis* essential oil was observed to be effective against *Dermanyssus gallinae* (Alimi et al. 2021). Organic solvents such as methanol, alcohol, ethyl acetate, petroleum ether, and acetone have been used to solubilize the acaricidal component(s) (Luo et al. 2015).

The present study revealed that ethyl acetate extracts of *U. fissa* including acaricidal component(s) at a concentration of 100 mg/ml killed all *S. scabiei* within 2 h, with an LC50 value of 19.14 mg/ml at 2 hours. Compared with other plant extracts, the acaricidal activity of *U. fissa* thus has certain advantages.

LC-MS analysis was used to evaluate the chemical composition of the ethyl acetate extract. This showed relatively high concentrations of total phenolics, flavonoids, and alkaloids. The primary constituents of the extracts were schaftoside (8.259%), carnosol (6.736%), prostaglandin A2 (5.94%), 13(*S*)-HpOTrE (4.624%), nandrolone (4.264%), 1*H*-indole-3-carboxaldehyde (4.138%), 9-oxoODE (3.206%), and stearidonic acid (2.891%). Several Chinese herbal remedies, including those from *Dendrobium nobile, Glycyrrhiza uralensis*, *Lysimachia christinae Hance*, *Rhizoma arisaematis*, and *Eleusine indica*, contain the flavonoid schaftoside (De Melo et al. 2005).

Many studies have observed that schaftoside has a variety of pharmacological effects, including the prevention of cholesterol gallstone disease, inhibition of glycosidases, and regulation of autophagy, as well as antioxidant, anti-obesity, anti-melanogenic, and anti-human respiratory syncytial virus activity (De Melo et al. 2005; Zhou et al. 2019a; Yi et al. 2022). Apigenin *C*-glycosides and other flavonoid glycoside chemicals, such as apigenin *C*-glycosides, can alter the feeding behaviour of the brown planthopper, resulting in its demise. Hao et al. (2018) demonstrated that schaftoside is markedly toxic to brown planthoppers.

Carnosol is an active polyphenolic component of *Rosmarinus officinalis *L., *Salvia fruticosa* Mill*,* and *Salvia officinalis* L. (Yang et al. 2022) with anti-inflammatory, anti-oxidant, anti-microbial, and anticancer properties (Alsamri et al. 2021). 1*H*-indole-3-carboxaldehyde is a key intermediate in the preparation of biologically active compounds and indole alkaloids (El-Sawy et al. 2017). The results of these studies show that the *U. fissa* extract contained a variety active ingredients. It is likely that the acaricidal activities of *U. fissa* may be attributed to the presence of terpenoids and flavonoids. Flavonoids and phenolics have been found to have multiple bioactivities (Alimi et al. 2021). However, the specific acaricidal components of the extract require further investigation. These preliminary results suggest that *U. fissa* may be a source of novel acaricidal substances capable of successfully controlling livestock mites. Additional systematic research is required to determine the active ingredients in *U. fissa* and assess them in clinical trials, acute toxicity tests on animals, and safety tests.

In conclusion, we assessed the effectiveness of *U. fissa* ethyl acetate extracts as an acaricide against *S. scabiei* and investigated the primary active components of the extract. The results have shown that the ethyl acetate extracts of *U. fissa* leaves have marked acaricidal activity *in vitro* and that the activity was both concentration- and time-dependent. The chemical composition of the ethyl acetate extract was determined by LC-MS analysis. This showed that the extracts contained significant amounts of active components.

Thus, ethyl acetate extracts of *U. fissa* offer a more cost-effective, safe, and environmentally friendly alternative to commercial medications for the management of arthropods hazardous to both human and animal health.

These extracts can create innovative biocides to protect crops and livestock. However, the precise active ingredients, clinical efficacy, and safety evaluations of these extracts require further investigation.
